# TNF-Like Weak Inducer of Apoptosis (TWEAK) Promotes Beta Cell Neogenesis from Pancreatic Ductal Epithelium in Adult Mice

**DOI:** 10.1371/journal.pone.0072132

**Published:** 2013-08-26

**Authors:** Fei Wu, Lili Guo, Aniela Jakubowski, Lihe Su, Wan-Chun Li, Susan Bonner-Weir, Linda C. Burkly

**Affiliations:** 1 Department of Immunobiology, Biogen Idec, Cambridge, Massachusetts, United States of America; 2 Section of Islet Cell and Regenerative Biology, Joslin Diabetes Center, Department of Medicine, Harvard Medical School, Boston, Massachusetts, United States of America; University of Bremen, Germany

## Abstract

**Aim/Hypothesis:**

The adult mammalian pancreas has limited ability to regenerate in order to restore adequate insulin production from multipotent progenitors, the identity and function of which remain poorly understood. Here we test whether the TNF family member TWEAK (TNF-like weak inducer of apoptosis) promotes β-cell neogenesis from proliferating pancreatic ductal epithelium in adult mice.

**Methods:**

C57Bl/6J mice were treated with Fc-TWEAK and pancreas harvested at different time points for analysis by histology and immunohistochemistry. For lineage tracing, 4 week old double transgenic mice CAII-CreER^TM^: R26R-eYFP were implanted with tamoxifen pellet, injected with Fc-TWEAK or control Ig twice weekly and analyzed at day 18 for TWEAK-induced duct cell progeny by costaining for insulin and YFP. The effect of TWEAK on pancreatic regeneration was determined by pancytokeratin immunostaining of paraffin embedded sections from wildtype and TWEAK receptor (Fn14) deficient mice after Px.

**Results:**

TWEAK stimulates proliferation of ductal epithelial cells through its receptor Fn14, while it has no mitogenic effect on pancreatic α- or β-cells or acinar cells. Importantly, TWEAK induces transient expression of endogenous Ngn3, a master regulator of endocrine cell development, and induces focal ductal structures with characteristics of regeneration foci. In addition, we identify by lineage tracing TWEAK-induced pancreatic β-cells derived from pancreatic duct epithelial cells. Conversely, we show that Fn14 deficiency delays formation of regenerating foci after Px and limits their expansion.

**Conclusions/Interpretation:**

We conclude that TWEAK is a novel factor mediating pancreatic β-cell neogenesis from ductal epithelium in normal adult mice.

## Introduction

Diabetes mellitusis manifested as hyperglycemia resulting from inadequate production of insulin by pancreatic β-cells. The ability of β-cells to expand or regenerate to restore adequate insulin production is limited, especially in adults. Attempts to increase β-cell mass through *de novo* pancreas regeneration or the expansion and differentiation of precursors *in vitro* for cell transplant therapy have been reported [1], [2], with limited success due to lack of understanding of the identity of β-cell progenitors and mechanisms regulating their fate in adults.

Numerous studies have determined that adult pancreas retains the intrinsic ability to make new insulin-producing β-cells, suggesting that facultative pancreatic progenitors exist within various cell types [3]. The conversion of mature α-cells to β-cells after extreme β-cell loss [4] and direct trans-differentiation from pancreatic exocrine cells to β-like cells after ectopic expression of *Ngn3*, *Pdx1* and *Mafa* [5] have been reported. Others reported that adult ductal epithelial cells can give rise to insulin-producing β-cells in rodents [6], [7], [8], recapitulating the role of ductal epithelium in embryonic development. After partial duct ligation (PDL), Ngn3-positive precursors in or near the ductal epithelium were found, suggesting activation of endocrine progenitors [9]. Ductal epithelium was definitively identified as the precursor for new endocrine cells and acinar cells both in neonates and after PDL in adults using the duct-specific carbonic anhydrase II (CAII) promoter to trace their fate [10]. However, in other lineage studies using Hnf1β or Sox 9 promoters, duct-derived endocrine cells were only observed during embryonic development [11], [12]. Further investigation is warranted to elucidate pathways promoting pancreatic β-cell regeneration in adults.

One such pathway may be tumor necrosis factor-like weak inducer of apoptosis (TWEAK), a unique TNF family cytokine that can regulate cell survival, growth, and differentiation, as well as inflammation and angiogenesis, through its cognate receptor FGF-inducible molecule 14 (Fn14) [13], [14]. Fn14 is expressed by non-hematopoietic cell types at relatively low levels in healthy tissues and dramatically upregulated locally within injured and diseased tissues [15]. Notably, substantial evidence has emerged that TWEAK can regulate progenitors expressing Fn14, including hepatic oval cells [16], skeletal muscle satellite cells and other mesenchymal lineage cell types.

Previously, we found that TWEAK expands liver progenitor cells as evidenced by hyperplasia of duct-like structures in liver of TWEAK-overexpressing mice [16]. Given the close developmental relationship between liver and pancreas, both arising from embryonic foregut endoderm [17], we hypothesize that TWEAK may exert similar effects on pancreatic ductal epithelium with progenitor potential.

## Materials and Methods

### Ethics Statement

All studies were carried out in strict accordance with the recommendations in the Guide for the Care and Use of Laboratory Animals of the National Institutes of Health. The protocol was approved by the Institutional Animal Care and Use Committee (IACUC) of Biogen Idec and Joslin Diabetes Center. All surgery was performed under anesthesia by i.p. injected ketamine/xylazine mixture (ketamine at 200 mg/kg b.w. and xylazine at 10 mg/kg b.w.), and all efforts were made to minimize suffering.

### Animals and Fc-TWEAK generation

C57Bl/6J and R26R-eYFP transgenic (Tg) reporter mice (Jackson Laboratory, Bar Harbor, ME); Fn14 knockout (KO) [18] and CAII-CreER^TM^ Tg mice [10] both on C57Bl/6 background were described previously. 8-week-old C57Bl/6 females, at least 4 per group, were injected intraperitoneally (i.p.) or subcutaneously (s.c.) with 200 μg mouse Fc-TWEAK, a fusion protein with the murine IgG2a Fc region [19] or mouse isotype control P1.17 once or twice weekly and then sacrificed at different time points. Aglycosylated Fc-TWEAK, agly-Fc-TWEAK, was constructed with the N297Q muIgG1 Fc sequence, thereby creating an Fc-effector function-deficient protein. For lineage tracing experiments, CAII-CreER^TM^ Tg mice [10] were mated with R26R-eYFP mice. Four week old double Tg (bigenic) were implanted with 3-week slow-release (1–2 mg/day) tamoxifen (TM) pellet (Innovative Research of America, Sarasota, Florida) followed by a 1 week washout period, and then injected with 200 μg Fc-TWEAK or control Ig twice weekly and sacrificed at day 18 after treatment; bigenic mice without TM, Fc-TWEAK or control Ig treatment were controls. Labeling efficiency is 30–40% [10].

### Tissue preparation and immunohistochemistry

Formalin-fixed human pancreas sections were from quality control blocks of cadaveric organs donated for islet isolation. Their use as exempt anonymous tissue had Joslin IRB approval. Fn14 immunostaining was as described [20].

Primary antibodies were: rabbit anti-pan-cytokeratin (CK) (1∶100), guinea pig anti-insulin (1∶150), rabbit anti-glucagon (1∶150), and rabbit anti-CD3 (1∶250) (Dako USA, Carpinteria, CA); rabbit anti-Ki-67 (1∶100, Vector Laboratories, Burlingame, CA), rat anti-F4/80 (1∶50, AbD Serotec, Raleigh, NC), humanized anti-Fn14 (P4A8 mAb, 2ug/ml [21], mouse anti-Ngn3 (1∶2000, Developmental Studies Hybridoma Bank, University of Iowa) guinea pig anti-human insulin (1∶200 Millipore, Billerica, MA) and rabbit anti-GFP (1∶4000, MBL International, Woburn, MA). Biotin-conjugated secondary antibodies were from Vector Laboratories. Antigen retrieval for Fn14 was performed with FICIN (Invitrogen, San Diego, CA) at 37 °C for 5 min, while for all others boiling in 0.1 M citrate buffer (pH6) followed by treatment with dual endogenous enzyme block (Dako). Primary antibodies, except Ngn3 and GFP, were incubated at room temperature for 1 hr.; Ngn3 and GFP were incubated overnight at 4°C and amplified using the tyramide system (Perkin Elmer, Waltham, MA). Anti-GFP to visualize YFP was pre-incubated overnight with poly-L-lysine (Sigma-Aldrich, St. Louis, MO) to remove non-specific staining due to charge [22]. TUNEL staining was performed with APOPTAG Peroxidase *in situ* Apoptosis Detection Kit (Chemicon, Temecula, CA).

### Partial pancreatectomy (Px)

Px was performed on C57Bl/6J mice as described for rats [6] with minor modification. CK-immunostained paraffin sections (two sections ≥200 μm apart per animal, 5–9 animals per phenotype) were examined. Regenerating foci were defined by their mesenchymal borders, with foci area determined by ImageScope (Aperio Technologies, Inc., Vista, CA), foci number determined by manual counting of all foci in the full pancreatic section, and foci stage defined as previously reported [8].

### Image analysis

For quantification of ductal proliferation, the number of Ki-67^+^ duct or duct adjacent cells and total duct cells were counted on one full footprint pancreatic section from at least 4 mice per time point and treatment. At least 300 total duct cells were counted. Cell types were defined by location and morphology: counted duct cells were the simple cuboidal cells of the interlobar and intralobar ducts, while ductal adjacent cells were single cells outside the duct epithelium but within the ductal stroma; small terminal ductules (squamous cells) were not included.

Quantification of Ngn3+ cells was performed on two full footprint pancreatic sections (≥200 μm apart) from each animal (4 animals per treatment); for each individual the average of the two sections was used. The total Ngn3+ cell number per section was manually counted and total pancreatic cell number per section counted by ImageScope, and these values were used to calculate the frequency of Ngn3+ cells.

For quantification of labeled cells in the lineage tracing experiment, one full footprint pancreatic section of each animal was scanned with manual counting at 10× for hormone staining and again for YFP staining. For YFP+ cells, higher magnification was used to check for co-expression. Hormone positive cells were categorized as islets (defined as more than 8 cells in cross section) and aggregates (defined as 8 cells or less in cross section).

### Fn14 mRNA expression

Mouse duct cells were isolated from islet-depleted tissue after collagenase digestion using immunomagnetic separation and anti-CD44 antibody. RNA from 3 independent samples (each 5–15 mice) was examined by qRT-PCR for Fn14 expression as described [16] with GAPDH used as internal control.

### Statistical analysis

Data are expressed as mean ± SEM and analyzed by 2-way ANOVA, with p<0.05 considered significant.

## Results

### TWEAK promotes proliferation of ductal cells in normal adult mouse pancreas through its receptor Fn14

**Figure 1:**
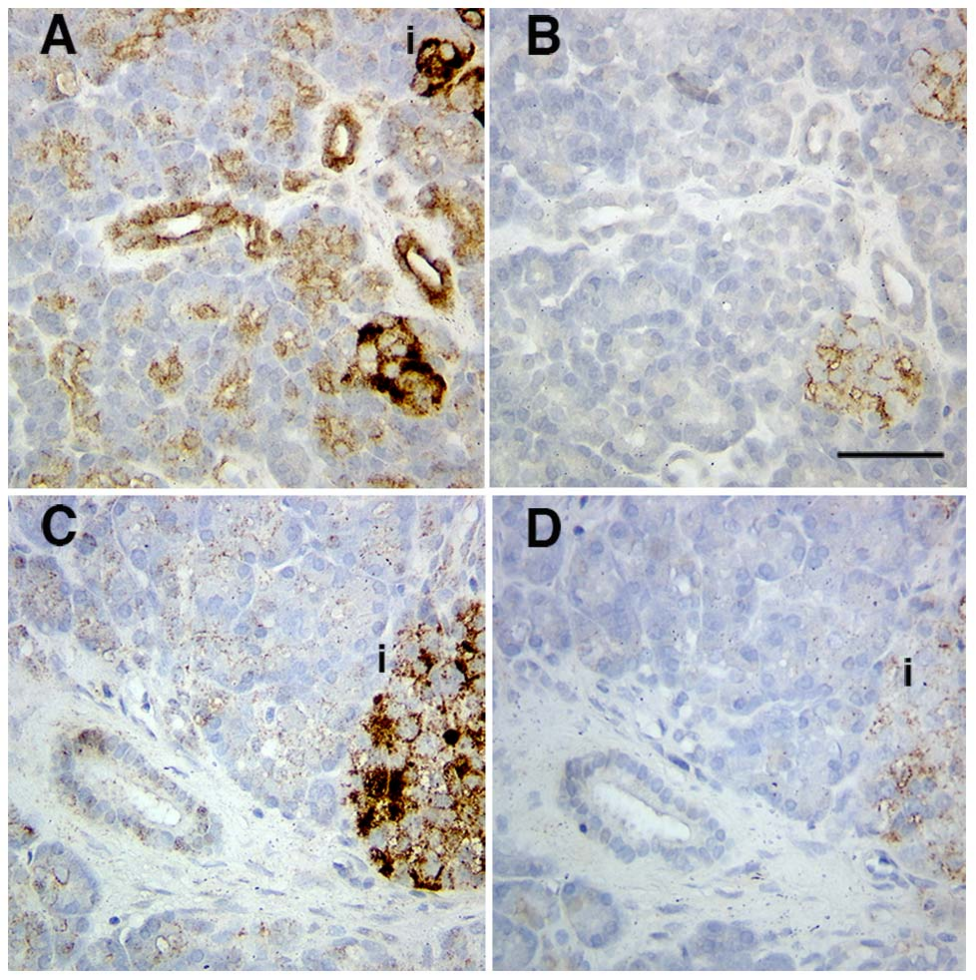
Fn14 expression by human pancreatic ductal epithelium. Variable amounts of Fn14 were seen in human pancreas with fairly strong intensity seen in tissue from a 16-year-old female donor (A) anti-Fn14 or (B) isotype control, and faint staining in a 57-year-old donor (C) anti-Fn14 and (D) isotype control. Specific staining was seen in islets as well as ducts. The islets were labeled as “i”. Scale bar  = 50 µm.

Immunostaining of human pancreas for Fn14 showed varying degrees of pancreatic duct epithelial staining in 3 of 4 human pancreas from organ donors without pre-existing pancreatic conditions. The strongest staining was observed in a young, obese donor with body mass index of 35 (Figure 1A–1B), whereas faint staining of ducts is shown in a 57-year-old donor (Figure 1C–1D). Donors also showed positive islet staining. Fn14 expression in the normal adult mouse pancreas was difficult to detect by immunostaining (data not shown) but was highly expressed by normal pancreatic duct cells at mRNA level (n = 3; values normalized to GAPDH were 0.014, 0.016, and 0.013, values comparable to positive control mouse lupus kidney samples); it is possible that Fn14 expression was upregulated during the isolation process.

**Figure 2:**
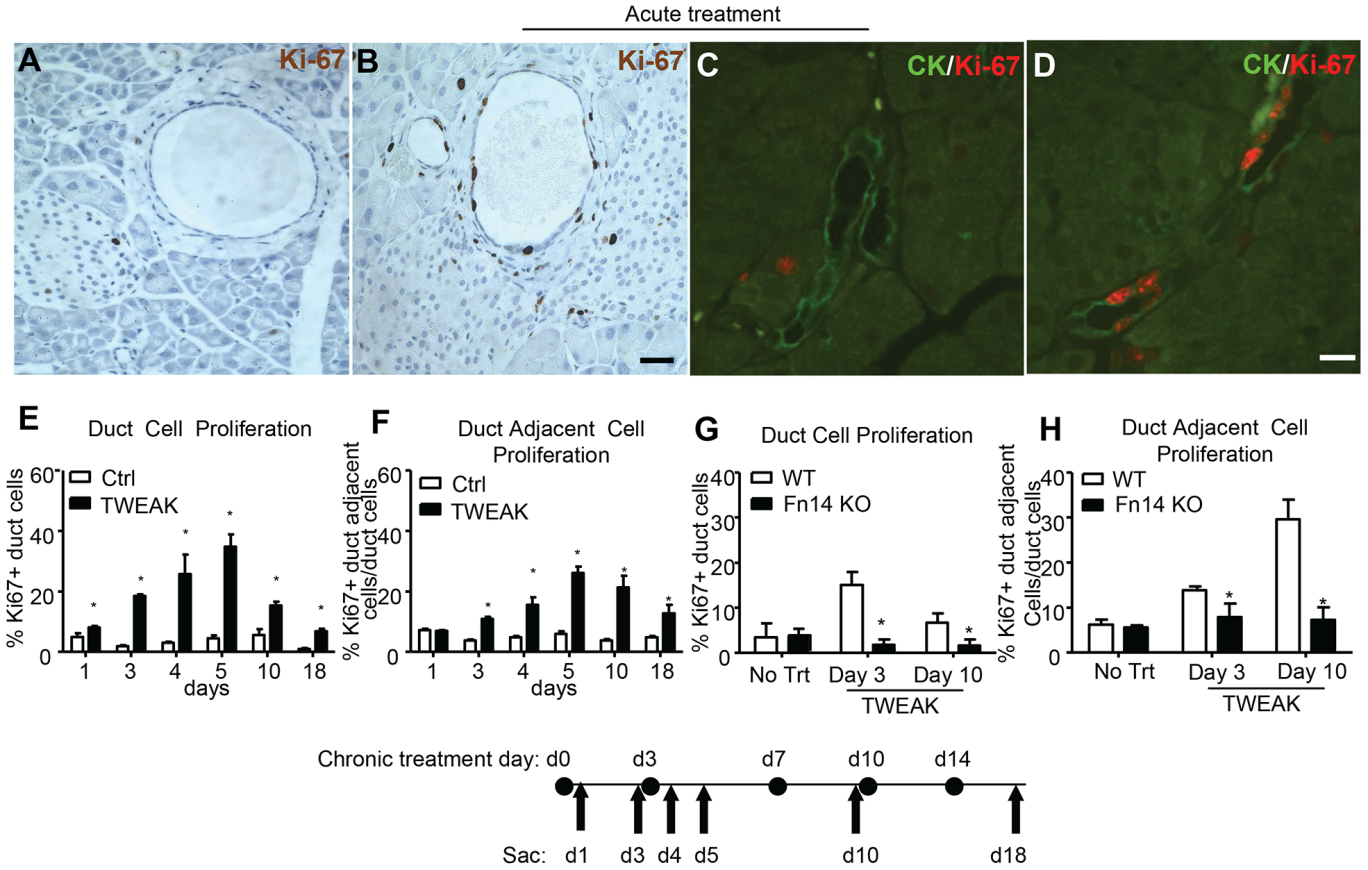
TWEAK treatment promotes proliferation of pancreatic duct and duct adjacent cells through its receptor Fn14. Pancreatic tissue of normal adult mice stained for Ki-67 (black) on day 3 after injection of (A) control Ig or (B) Fc-TWEAK, and costained with Ki-67 (red) and ductal epithelial marker CK (CK) (green) on day 4 after (C) control Ig or (D) Fc-TWEAK acute treatment. Scale bar  = 10 µm. (E–F) Quantification of the % Ki-67^+^ pancreatic (E) duct and (F) duct adjacent cells in mice treated with control Ig or Fc-TWEAK twice weekly. Data are shown as mean±SEM (n = 4). * p<0.05 for Fc-TWEAK vs control Ig treatment. (G–H) Quantification of the % Ki-67^+^ (G) duct and (H) duct adjacent cells in normal adult WT or Fn14 KO mice treated with control Ig or Fc-TWEAK twice per week. Data are shown as mean±SEM (n = 4) for percentage of Ki-67+ duct cells (E&G) or Ki-67+ duct adjacent cells per total duct cells (F&H); * p<0.05 for Fc-TWEAK treatment vs control Ig.

We investigated the effect of TWEAK overexpression on pancreatic ductal cells by Fc-TWEAK injection in normal adult mice. Increased numbers of Ki-67+ pancreatic duct epithelial cells and adjacent cells were observed three days after a single injection *(acute treatment)* of Fc-TWEAK compared to control Ig (Figure 2A–2B). Co-staining for Ki-67 and pan-cytokeratin (CK), a duct epithelial cell marker (Figure 2C–2D) confirmed the ductal identity of the proliferating cells. We determined the kinetics of the mitogenic effect of TWEAK in mice treated twice weekly for two weeks *(chronic treatment)*. Duct cell proliferation was detected as early as day 1 after exposure and peaked between days 4–10, although significant proliferation was still detected at day 18 (Figure 2E). Proliferation of duct adjacent cells was somewhat later, with a significant increase first apparent at day 3 after Fc-TWEAK treatment, and sustained relatively longer (Figure 2F). We did not observe any mitogenic effect of TWEAK on the acinar cells or islet cells at any of these time points. The kinetics of proliferation after acute Fc-TWEAK followed a similar but less prolonged pattern as compared to chronic treatment (Figure S1A–S1B).

Fc-TWEAK induced robust proliferation of duct and duct adjacent cells in WT but not Fn14 KO mice (Figure 2G–2H), clearly indicating that the mitogenic effect of TWEAK on duct cells is dependent upon Fn14. These data suggest that TWEAK acts directly on Fn14 expressed by pancreatic duct epithelium to induce proliferation.

TWEAK has proapoptotic and proinflammatory effects in some contexts [23], [24], which raised the possibility that the increased ductal proliferation might be a secondary effect. To rule out these possibilities, apoptosis and immune cell infiltration were assessed. Little TUNEL, CD3 or F4/80 positive staining was seen in the pancreas of animals treated with control Ig, indicating that few apoptotic cells, T cells or macrophages are normally present. Similarly, at all time points studied (day 1, 3, 4, 5, 10 and 18), there were no changes in TUNEL signal (represented in Figure S2), and no generalized changes in CD3 or F4/80 expression (Figure S3) after twice weekly Fc-TWEAK as compared to control Ig-treatment. These data indicate that the increased proliferation of duct and duct adjacent cell induced by TWEAK was not due to increased cell turnover or generalized tissue inflammation.

To rule out the possible involvement of Fc-effector functions, we treated mice twice weekly with agly-Fc-TWEAK, and examined cell proliferation at day 5 after treatment (Figure S4A–4B). Similar duct cell proliferation was observed after agly-Fc-TWEAK and Fc-TWEAK treatment, therefore Fc-effector function is not required. We also treated mice by subcutaneous instead of intraperitoneal injection to rule out an effect specific to route of delivery (Figure S4C–4D).

### TWEAK induces endogenous Ngn3^+^ cells in normal adult mouse pancreas

**Figure 3:**
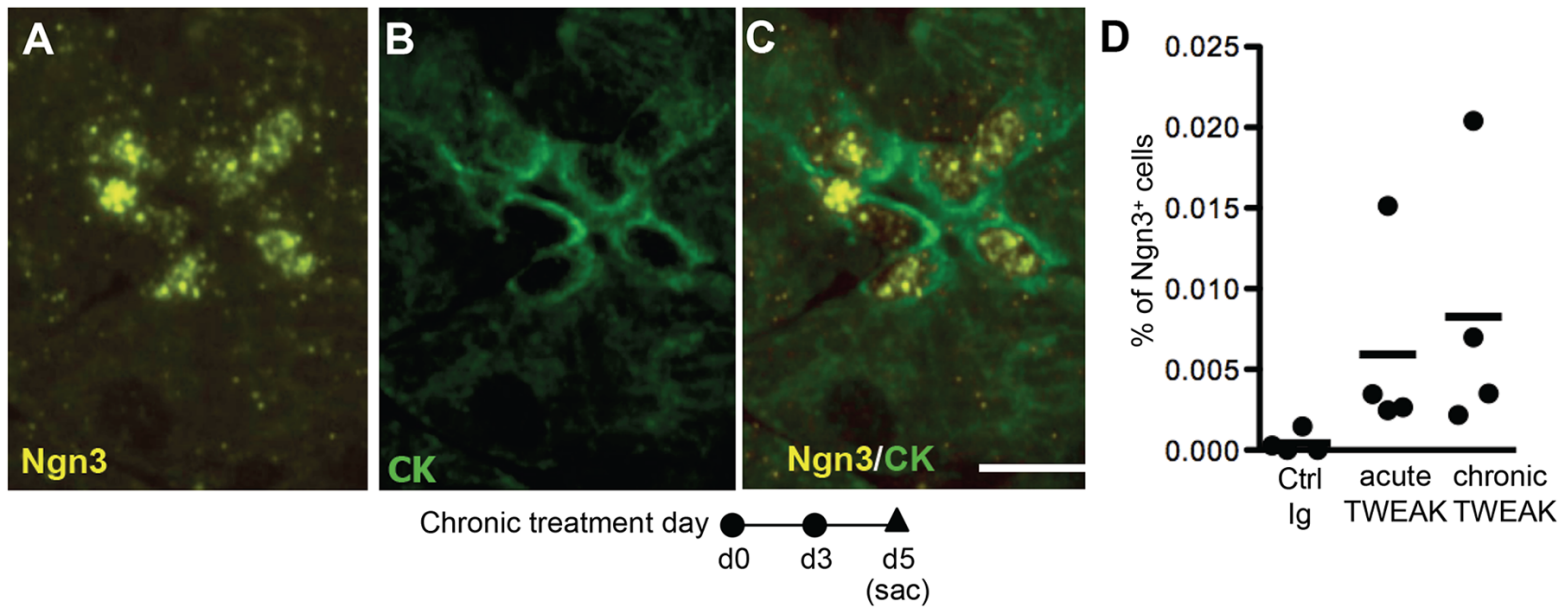
TWEAK treatment induces Ngn3 expression in pancreatic duct cells. Immunostaining for (A) Ngn3 (yellow), (B) CK (green) and (C) Ngn3/CK (shown as merge) on day 5 of chronic TWEAK treatment. Scale bar  = 10 µm. (D) Quantification of Ngn3^+^ cells on day 5 after twice weekly control or TWEAK treatment. Each point represents an individual animal, with horizontal bar the mean of 4 mice/group.

Fc-TWEAK robustly induced proliferation of duct cells, so we investigated its effect on induction of the endocrine progenitor marker Ngn3 [25]. No Ngn3 expression was detected in pancreas from control Ig-treated mice. However, at day 4 or 5 after twice weekly Fc-TWEAK treatment (Figure 3A–C), Ngn3 expression was mostly detected in small ducts (CK+), centroacinar cells and duct adjacent cells (Ngn3^+^ CK^−^) still in contact with CK^+^ duct cells; Ngn3+ cells did not express any islet hormones (data not shown). These Ngn3+ cells were not distributed evenly over the whole section but tended to form clusters as shown in Figure 3. Ngn3 was only detected at day 4 or 5 and numbered only a few per ten thousand adult pancreatic cells (Figure 3D), which is consistent with transient expression of Ngn3 shown in embryonic development [26], [27], [28].

### Chronic TWEAK treatment induces focal ductal structures with characteristics of regenerating foci in normal adult mice

**Figure 4:**
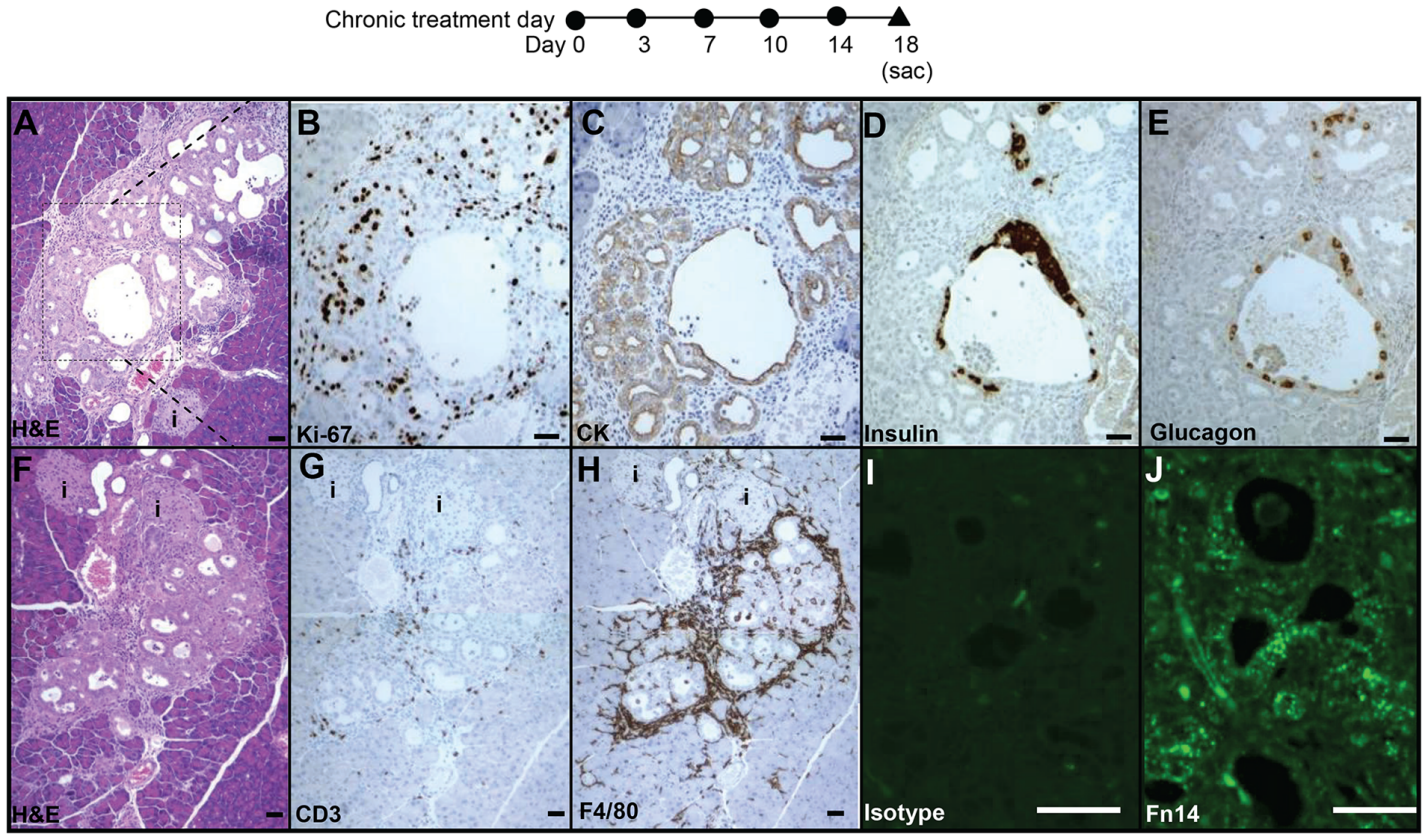
Chronic TWEAK treatment mimics the response to the partial pancreatectomy. Ductal structures with characteristics of regenerative foci were identified by H&E staining (A) at day 18 after TWEAK treatment twice weekly in all 4 treated animals. Inset of (A) is shown on serial sections stained with Ki-67 (B), CK (C), insulin (D) and glucagon (E). (F–H) Serial sections of area from another representative animal were stained with H&E (F), CD3 (G) F4/80 (H); isotype control (I) and Fn14 (J). The islets were labeled as “i”. Scale bar  = 50 µm in all panels.

Given the appearance of Ngn3 expression in normal adult mouse pancreas at day 5 after Fc-TWEAK treatment, we investigated the effects of longer term TWEAK treatment. At day 18 after chronic Fc-TWEAK but not control Ig treatment, we observed the appearance of focal areas characterized by well-defined structures of proliferating ductules surrounded by mesenchymal cells, with some hormone-expressing cells (insulin^+^ or glucagon^+^) within the duct epithelium as well as scattered hormone-expressing cells (Figure 4A–4E). Hormone^+^ cells budding from proliferating ductules suggest that they may be newly formed and have not yet migrated to form islets. Consistent with this notion, some of these insulin+ cells had strong significant pan-CK immunostaining (Figure S5. No bihormonal insulin+glucagon+ cells were seen.

These focal areas induced by TWEAK treatment are similar to those transiently found in adult rats after Px, which have been shown to form new pancreatic lobes [6], [8], [29], [30]. In addition, concentrated CD3^+^ or F4/80^+^ cells were restricted to these TWEAK-induced focal areas (Figure 4G–4H) and not generalized throughout the rest of the pancreas (Figure S3) at day 18 after chronic treatment, much as in the focal regions of regeneration after Px but not the remnant pancreas (Figure S6). For each animal, we examined over 10 full footprint pancreatic sections (≥ 200 μm apart) representing the entire pancreatic block. The incidence of ductal structures with characteristics of regenerating foci was low, with generally only one large area per pancreatic section from each Fc-TWEAK-treated mouse; no such foci were found in sections from control-Ig treated mice.

Notably, Fn14 protein expression was difficult to detect in untreated mice (data not shown) but readily detectable in the ductal structures in these focal areas (Figure 4J) and more strongly expressed than in ducts outside these areas. Thus our data suggest that the observed areas of ductal structures with characteristics of regenerating foci originated from pancreatic duct epithelial cells that were induced by TWEAK to proliferate and expand.

### TWEAK induces pancreatic β-cells from duct epithelium

**Figure 5:**
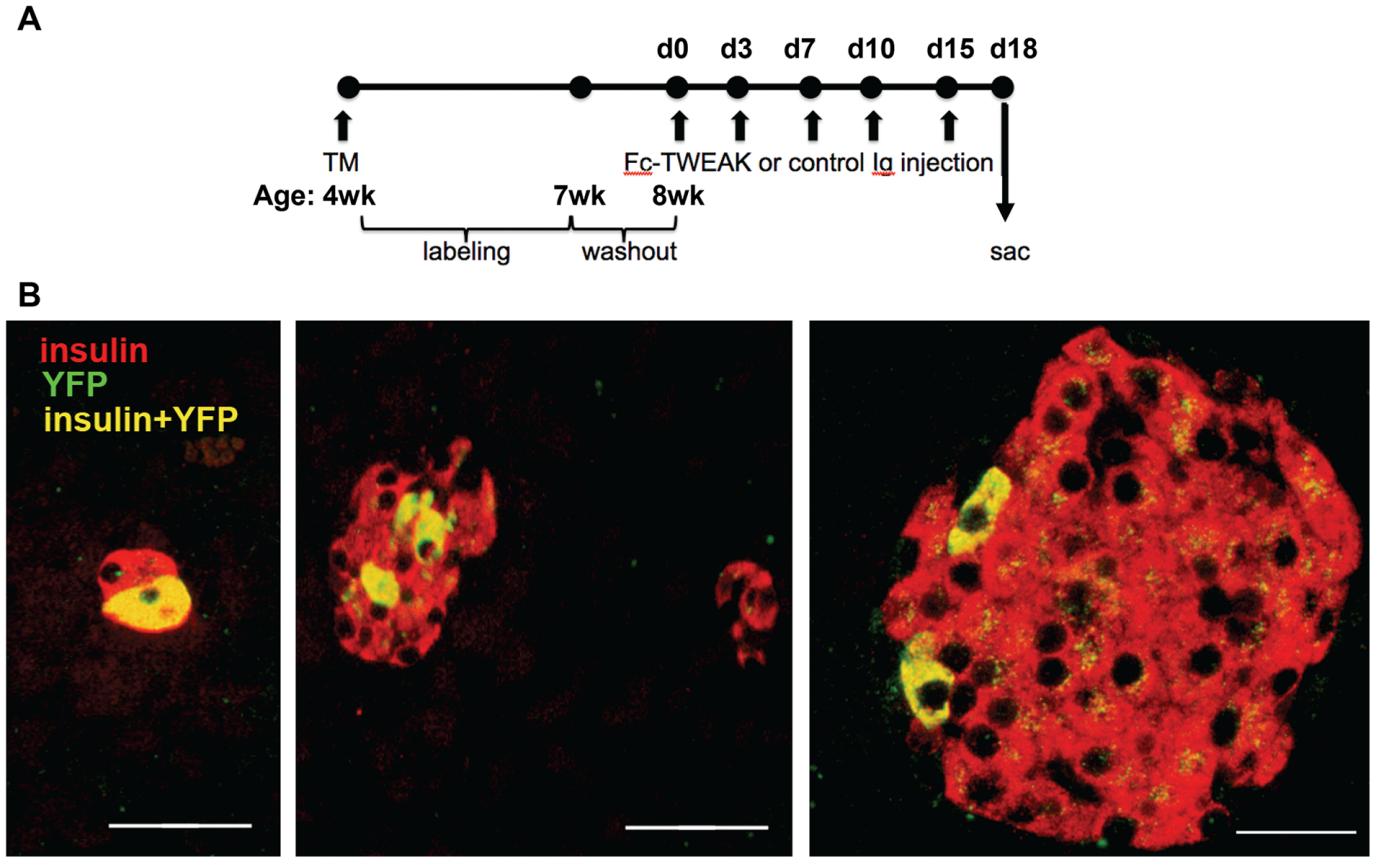
TWEAK promotes pancreatic regeneration through activation of pancreatic duct-derived progenitor cells. (A) CAII-CreER^TM^:R26R-eYFP (bigenic) mice were implanted with 3-week slow-release TM pellets at 4 weeks of age, followed by a 1 week washout period, and received treatment at 8 weeks of age twice weekly for 2 weeks, and killed at 18 days after start of treatment. (B) In sections from TM-treated mice immunostained for YFP (green) and insulin (red), YFP-marked insulin+ (coexpression, yellow) cells could be found in small aggregates and within islets. In control bigenic animals without tamoxifen, control Ig or Fc-TWEAK treatment, no labeled cells were found. Scale bar  = 50 µm in left two panels and 20 µm in far right panel. Quantification of labeled islets and aggregates is presented in Table 1.

To identify the progeny of TWEAK-induced proliferating duct cells, we used the CAII-CreER^TM^:R26R-eYFP (bigenic) mice for lineage tracing. In the TM-treated bigenic mice, YFP-marked insulin+ cells were found in 21% of islets and 3.9% aggregates after chronic Fc-TWEAK treatment but only in 1.2% aggregates after control Ig treatment (Figure 5A and Table 1). Of interest, the labeled cells were most often seen as a few cells within islets (Figure 5B). As an additional control to rule out possible induction of CAII by TWEAK, MIN6 cells were treated for 24 and 48 hrs with TWEAK. QPCR analysis showed that TWEAK did not induce an increase of the extremely low level of CAII mRNA expressed in this murine insulin expressing cell line (Figure S7).

**Table 1:** Quantification of YFP marked insulin^+^ cells in CAII-CreER^TM^:R26R-eYFP (bigenic) mice 18 days after chronic Fc-TWEAK or control Ig treatment.

Treatment group	N (animals)	# of YFP^+^ islets /animal (%)	Total islet # counted/ animal	# of YFP^+^ aggregates/ animal (%)	Total aggregate #/ animal	YFP^+^ cells in islets/ animal	YFP^+^ cells in aggregates/ animal
**Control (no TM & drug treatment)**	5	0	41.0±9.4	0	67.6±10.5	0,0,0,0,0	0,0,0,0,0
**TM + control** **Ig**	6	0	45±5.8	1.0±0.3 (1.2)	81.3±14.9	0,0,0,0,0,0	1, 0, 2, 1, 1, 4
**TM + Fc-** **TWEAK**	5	10±5.4 (21)	47±5.0	2.2±1.3 (3.9)	56.0±4.6	4, 14, 9, 194, 18	0,3,1,19,0

All animals were bigenic CAII-CreER^TM^:R26-eYFP mice. Control mice had no TM or drug (control Ig or Fc-TWEAK) treatment. For each animal, all insulin positive cells on a single full footprint pancreatic section were examined for co-expression with eYFP in islets or in aggregates (defined as being 8 cells or less in cross section). Mean values ±SEM are reported, and the last two columns report individual values/animal.

### Fn14 deficiency delays pancreatic regeneration after Px

**Figure 6:**
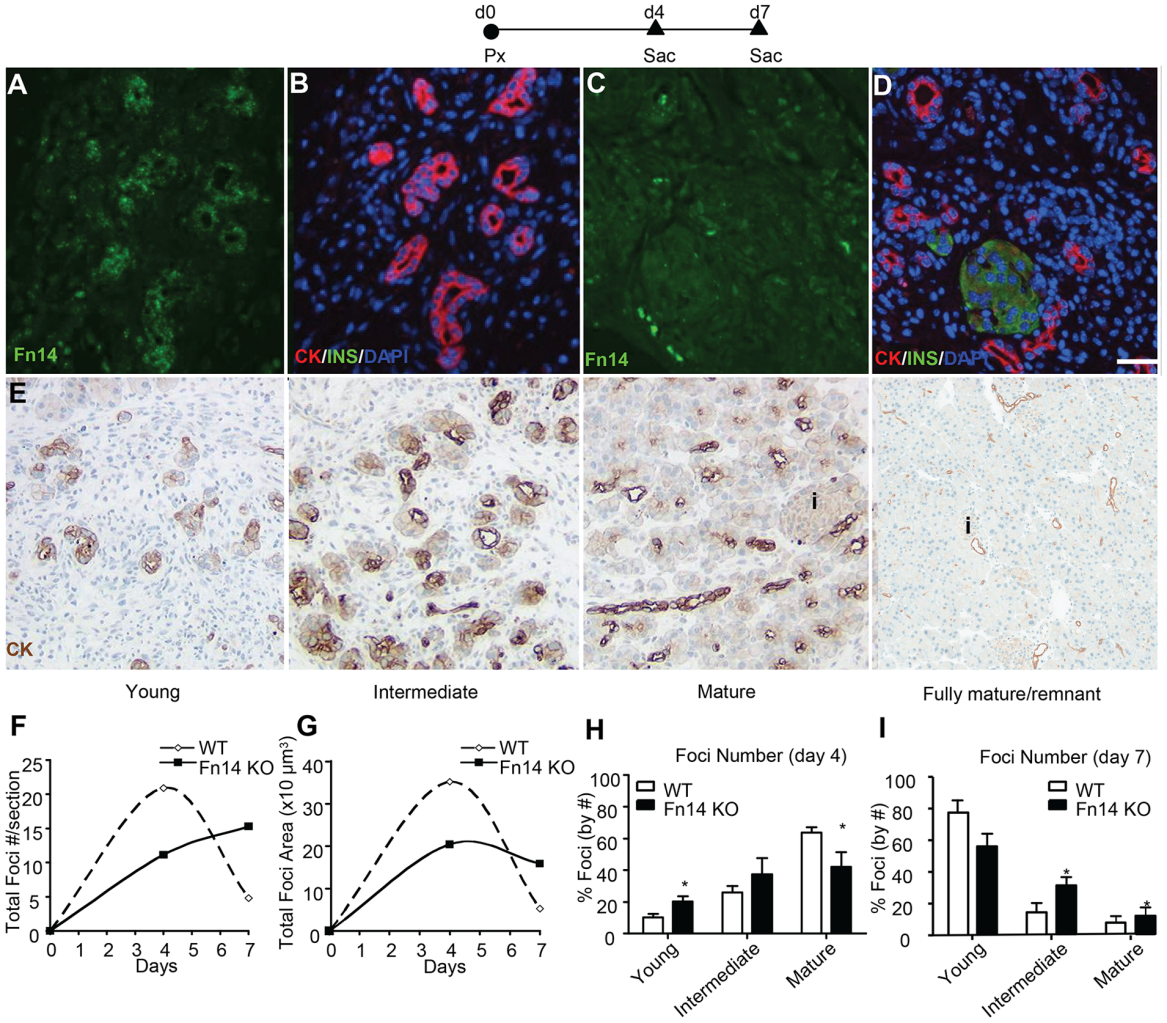
Fn14 is transiently expressed in the regenerating foci after Px and its deficiency delays pancreatic regeneration. (A–D) Representative images from serial sections immunostained for Fn14 (green) (A, C) and CK (red)/insulin (green)/DAPI (blue) (B, D) show transiently enhanced expression of Fn14 in immature regenerating foci (A, B) but not in mature foci (C, D). E. Representative stages of the maturation of the regenerating foci as previously defined [11]. (F–G) Quantification of (F) total foci number and (G) total foci area per section of Fn14 KO mice or WT mice at day 4 and day 7 post surgery. (H–I) Distribution of foci at different stages of differentiation in Fn14KO and WT mice at (H) day 4 or (I) day 7 post surgery. Number and stage of regenerating foci were assessed on at least two sections/animal (≥ 200 µm apart) in WT and Fn14 KO mice (n ≥ 5 per group). No regenerating foci were identified in sham-operated mice. Data are shown as mean±SEM; * P<0.05 for Fn14 KO vs WT mice.

Since our studies have shown that TWEAK overexpression induces pancreatic β-cell neogenesis in normal adult mice, we asked whether the endogenous TWEAK/Fn14 pathway plays a role in pancreatic regeneration. Fn14 is dramatically increased in injured and diseased tissues [15], so we investigated whether Fn14 expression was upregulated in the Px model. Previously we defined three stages of regenerating foci after Px [8]: young, intermediate, and mature stage foci, as illustrated in Figure 6E with CK staining. Fn14 was clearly expressed at relatively high levels in the proliferating ductules of immature regenerating focal areas 4 days after Px but not in mature regenerating focal areas in which there were already differentiated acinar and islet cells (Figure 6A–6D).

Pancreatic regeneration was compared between WT and Fn14 KO mice by assessment of the number of regenerating foci, total area of the foci and their maturity at several time points after surgery. WT mice exhibited increased number and total area of foci on day 4, which declined by day 7. In contrast, Fn14 KO mice had different kinetics and extent of regeneration, with a delay in increased number and decreased area of foci of regeneration compared to WT (Figure 6F–6G). Thus Fn14 deficiency both delayed the initiation of foci formation (as seen by the decreased number of foci at 4 days in Fn14 KO compared to WT mice) and limited their expansion (as seen by the smaller total area of foci). A difference in WT vs KO mice was also observed in foci maturation. At 4 days post surgery, WT mice had a greater proportion of mature foci than young foci. Yet by day 7, the distribution has shifted in favor of young foci, presumably because the mature foci reached a stage at which they are no longer distinguishable from remnant tissue and therefore not counted. In contrast Fn14 KO mice at day 4 had a greater proportion of younger stage regenerating foci (Figure 6H) and even by day 7 post surgery, their maturation still lagged behind that of WT, with a higher proportion of intermediate foci and a trend to more mature stage foci (Figure 6I). Taken together, these data indicate that disruption of Fn14 signaling delays initiation of formation of regenerating foci and limits their expansion as compared with WT mice.

### Proposed model (Figure 7)

In this study, we demonstrated that in normal adult pancreas, *in vivo* TWEAK treatment promotes duct cell proliferation, induces Ngn3 expression and focal duct structures with characteristics of regenerating foci. The cell precursor/progeny relationship was addressed by lineage tracing, proving that duct epithelial cell progenitors give rise to pancreatic β cells in response to TWEAK. Furthermore, we demonstrated a role for endogenous TWEAK in promoting pancreatic regeneration after Px.

**Figure 7:**
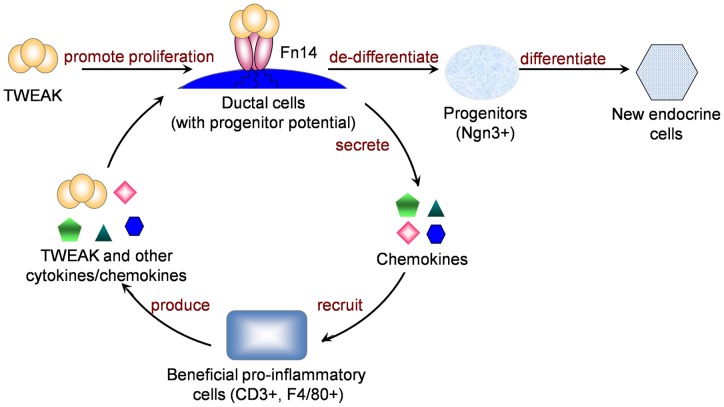
Proposed Model for TWEAK’s effect on pancreatic regeneration. TWEAK signals through Fn14, which is normally expressed at low levels in adult pancreatic ducts and upregulated after injury, resulting in proliferation of duct epithelium followed by differentiation to mature pancreatic cell types, including β-cells. In addition, TWEAK/Fn14 signaling induces a localized leukocyte infiltrate that may be beneficial to the tissue regeneration, thereby creating a positive feedback loop. Although not addressed in the current study, this likely occurs through induction of chemokines or cytokines that promote inflammatory cell recruitment. We speculate that the proliferating duct epithelial cells regress to a less differentiated state with progenitor potential, giving rise to Ngn3+ endocrine progenitors that then differentiate to new endocrine cells.

## Discussion

In this report, we identify TWEAK as a novel pancreatic β-cell neogenesis factor, demonstrating that it promotes β-cell formation from pancreatic duct epithelium in normal adult mice. Importantly, we also show that the endogenous TWEAK/Fn14 pathway plays an important role in regulating pancreatic regeneration in the Px model.

Studies have shown various sources of new pancreatic β-cells in different models of injury [9], [10], [11], [12]. While there is some controversy, multiple reports support that pancreatic duct cells have progenitor potential that is activated after injury [1], [6], [7], [8], [10], recapitulating their role in embryonic development [31]. Molecular pathways regulating this process have been identified through KO mice [25], [32], and several pharmacological approaches have been pursued for β-cell regeneration, including GLP-1 receptor agonists [33], [34], gastrin [35], [36], growth factors including TGFα [37], KGF [38], betacellulin [39], islet neogenesis-associated protein (INGAP) [40], and the cytokine IFNγ [7]. However, none of these studies proved by lineage tracing the cell of origin of *de novo*-generated β cells. Our studies contribute new findings, that TWEAK acts as a novel pancreatic β-cell neogenesis factor through activation of ductal epithelial progenitor cells in normal adult mice.

The neogeneic effect of TWEAK is initially manifested by the proliferation of duct epithelial cells, consistent with its capacity as a mitogenic factor for the progenitorss in other tissues [15]. We also observed proliferating duct adjacent cells, which did not express duct epithelial marker CK but were still in contact with the duct epithelium. These duct adjacent cells were not acinar, islet or inflammatory cells. The kinetics of their proliferation lagged slightly behind that of the ductal epithelial cells suggesting the intriguing possibility that they were delaminated ductal epithelial cells migrating away from the duct; however we cannot rule out that they are mesenchymal cells. Our observation that the endocrine progenitor cell marker Ngn3 was expressed by both the duct and duct adjacent cells also supports that these cells have islet progenitor cell potential. While the fate of these Ngn3+ cells was not formally addressed in our study, we definitively prove that the duct epithelial cells were the progenitors of β-cells after chronic TWEAK treatment based on the appearance of YFP+/insulin+ cells in a ductal epithelium lineage tracing system. Interestingly, we found that the labeled cells were most often seen as a few cells within islets and only occasionally as aggregates. This finding may appear at odds with our observation of insulin+ cells in the focal ductal structures shown at the same timing. However, given that those ductal structures were observed at a low frequency, it is not surprising that our fate mapping result represents the balance of the tissue. We speculate that the proliferating labeled duct and duct adjacent cells migrated and coalesced into islet cell clusters and pre-existing islets. We found YFP+/glucagon+ cells within islets, although less frequently than the YFP+/insulin+ cells (data not shown), presumably reflecting the lower frequency of α-cells as compared to β-cells. Since the labeling efficiency of this lineage tracing mouse strain is around 30%, the regenerative effect of TWEAK may be underestimated in our study. The recent report that tamoxifen can induce Cre-loxP recombination in pancreatic islets in adult mice long after administration due to its retention in the body complicates all lineage tracing experiments using the Cre-LoxP system. [41]. However, since in our mouse model the transgene CAII and Cre are expressed in murine ductal but not in murine islet β-cells [10] and as we showed Tweak does not induce CAII in β-cells, such retention if it occurred would only label the ducts.

Our studies show that the regenerative activity of TWEAK in the adult pancreas is dependent on its receptor Fn14. Our data suggest that TWEAK may act directly through Fn14 expressed on duct epithelial cells to induce their proliferation as an early step in the neogeneic process and is consistent with the lack of proliferative effect of TWEAK on acinar and islet cells in our study. Enhanced Fn14 expression was detected on focal duct structures characterized by extensive proliferation induced after 18 days chronic TWEAK treatment. Fn14 expression was also increased after Px, consistent with its induced expression after injury. Notably, the increased Fn14 expression after Px is only observed transiently in immature foci, not mature foci, again consistent with an important role early in the process of pancreatic regeneration. In human pancreas, various levels of Fn14 protein were observed. As in normal adult mice, several human donors showed only faint Fn14 immunostaining. Interestingly, relatively high Fn14 expression was observed on duct cells that were prominent in the pancreas of a young, obese donor, suggesting that it may be induced as a compensatory mechanism in this condition. Although we detect Fn14 expression in human islets, this could not be addressed in mice since their islets had no apparent Fn14 expression; this will be a point of future study.

Regenerating foci have been observed after Px [3], [6], [8], [29] and in other models of β-cell regeneration, including PDL [42], transgenic mice overexpressing interferon γ (IFN-γ) on the human insulin promoter [7], rats with prolonged glucose infusion [43] and spontaneous autoimmune BB-rats [44]. They are well-defined structures consisting of abundant fibroblast-like stromal cells, increasingly branched ductules, and eventually acinar and islet cells that form new lobes of pancreas indistinguishable from the original pancreatic tissue. After Px, proliferation of cells within the common pancreatic duct [6], [8] is followed by their de-differentiation/expansion and the transient appearance of regeneration foci. Importantly, we demonstrated that overexpression of a single factor, TWEAK, can induce the appearance of similar structures as those in the response to Px.

We also demonstrated for the first time that the endogenous TWEAK/Fn14 pathway can regulate pancreatic regeneration. Fn14 deficiency delayed pancreatic regeneration after Px, with similar results obtained in the TWEAK KO mouse (data not shown). The mechanism of action of TWEAK/Fn14 is early in the regeneration process, with delayed initiation and limited expansion of the regeneration foci in the KO mice. The delay in foci maturation is most likely secondary to the delays in these early events.

Specialized phagocytes also play an important role in tissue development and regeneration [45]. Macrophages are important in fetal pancreatic development, especially for ductal branching [46], as are phagocytes in regenerative responses to tissue injury in other organs [18] and [47]. Regenerating foci after Px and similar focal structures induced by chronic TWEAK administration in normal adult mice also contain local immune infiltrates, especially F4/80+ macrophages, but notably, chronic TWEAK treatment did not induce generalized inflammation. As the function of the local infiltrates was not directly addressed, we cannot rule out their contribution to pancreatic regeneration, possibly by release of cytokines and interaction with other non-inflammatory cells in the immediate vicinity.

In conclusion, our study sheds new light on the regulation of pancreatic β-cell regeneration with identification of TWEAK/Fn14 as a novel pathway promoting neogenesis in normal adult animals from pancreatic ductal epithelium. Furthermore, we identify TWEAK overexpression as a valuable tool to elucidate molecular and cellular pathways of β-cell regeneration and as a potential means to pharmacologically promote pancreatic β-cell regeneration for diabetes treatment.

## Supporting Information

Figure S1 Acute TWEAK treatment promotes proliferation of duct and duct adjacent cells. Quantification of the % Ki-67+ duct (A) and duct adjacent cells (B) per total duct cells in pancreas from normal adult mice at various time points after single injection of TWEAK injected on day 0. Data are shown as mean±SEM (n = 4); * P<0.05 for TWEAK treatment vs control.
(TIFF)

Figure S2 TWEAK treatment does not induce apoptosis in pancreas. Sections of pancreas from mice treated twice per week with control (A) or TWEAK (B) were assayed for TUNEL signal and no increase was seen at any of the time points, examined day 1, 3, 4, 5, 10 and 18; representative sections shown from day 18 after treatment. (C) Lymph node as a positive control of TUNEL staining. Scale bar  = 100 μm.
(TIFF)

Figure S3 TWEAK treatment does not induce generalized pancreatic inflammation. Serial sections of pancreas from normal adult mice treated twice weekly with control (A, D) or TWEAK (B, E) immunostained for CD3 (A, B) and F4/80 (D, E) on day 18 after treatment. Positive control staining in spleen for CD3 staining (C) and in liver for F4/80 staining (F). Scale bar  = 50 μm.
(TIFF)

Figure S4 TWEAK-induced duct cell proliferation does not involve Fc-effector function nor is caused by peritonitis. (A–B) Quantification of % Ki-67+ duct (A) and duct adjacent cells (B) per total duct cells in pancreas from normal adult mice at day 5 after Ctrl mIgG2a (P1.17, control for Fc-TWEAK), Fc-TWEAK, Ctrl mIgG1 (1E6, (control for Agly-Fc-TWEAK) or Agly-Fc-TWEAK twice weekly. Controls (white bars); TWEAK treated (black bars). (C–D) Quantification of the % Ki-67+ duct (C) and duct adjacent cells (D) per total duct cells in pancreas at day 5 after Crtl (control Ig P1.17) or Fc-TWEAK subcutaneously (s.c.) or intraperitoneally (i.p.). Controls (white bars); TWEAK treated (black bars). Data are shown as mean±SEM (n = 4); * P<0.05 for TWEAK treatment vs control.
(TIFF)

Figure S5 Co-expression of insulin and pan-cytokeratin in some cells of the focal ductal structures after chronic TWEAK treatment. Immunofluorescent staining of pancreas at day 18 after chronic TWEAK treatment (A) shows some insulin positive cells (red) also express panCK (green) (coexpression- yellow/orange) in the complex ductal structures but the Ig controls (B) do not have these regions and have no co-expression of the insulin and panCK. Magnification bar  = 50 µm.
(TIFF)

Figure S6 Inflammatory infiltrates were recruited to the regenerating foci after Px. Serial sections from sham-operated (A, B) and Px at 4 days post surgery (C, D) were immunostained for CD3 (A, C) and F4/80 (B, D). Scale bar  = 200 μm.
(TIFF)

Figure S7 Culture of MIN6 cells with TWEAK does not induce CAII mRNA. Q-PCR of MIN6 cells treated for 24 or 48 hr with Tweak (100 ng/ml). Data were normalized to ribosomal S18 and expressed relative to time zero. Data are shown as mean±SEM from 4 independent experiments.
(TIFF)
