# Supplementary figures and images for: TNF-Like Weak Inducer of Apoptosis (TWEAK) Promotes Beta Cell Neogenesis from Pancreatic Ductal Epithelium in Adult Mice
Source: PLoS One. 2013 Aug 26;8(8):e72132. doi: 10.1371/journal.pone.0072132 (PMC3753348; doi:10.1371/journal.pone.0072132)

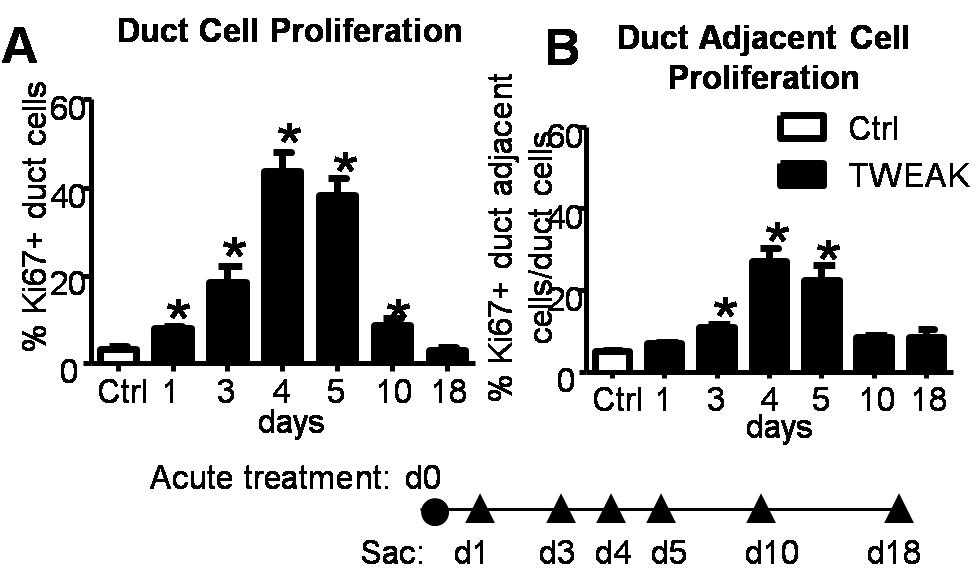

Supplement: Figure S1 — Acute TWEAK treatment promotes proliferation of duct and duct adjacent cells. Quantification of the % Ki-67+ duct (A) and duct adjacent cells (B) per total duct cells in pancreas from normal adult mice at various time points after single injection of TWEAK injected on day 0. Data are shown as mean±SEM (n = 4); * P<0.05 for TWEAK treatment vs control. (TIFF) [file pone.0072132.s001.tif]

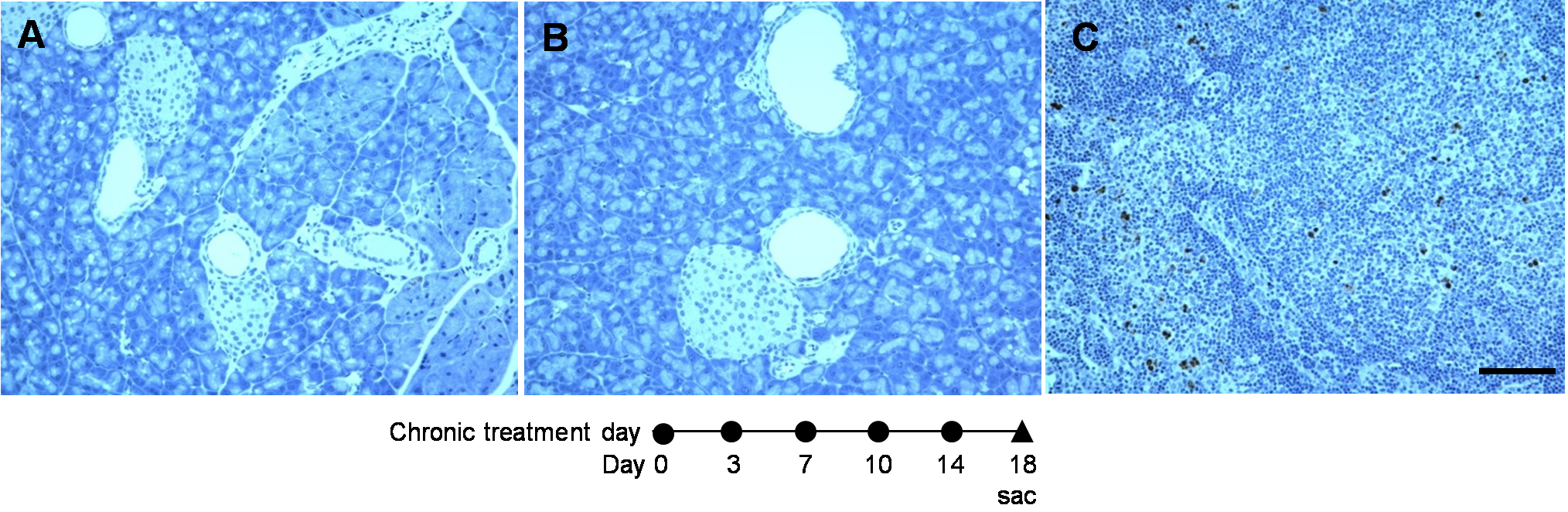

Supplement: Figure S2 — TWEAK treatment does not induce apoptosis in pancreas. Sections of pancreas from mice treated twice per week with control (A) or TWEAK (B) were assayed for TUNEL signal and no increase was seen at any of the time points, examined day 1, 3, 4, 5, 10 and 18; representative sections shown from day 18 after treatment. (C) Lymph node as a positive control of TUNEL staining. Scale bar = 100 μm. (TIFF) [file pone.0072132.s002.tif]

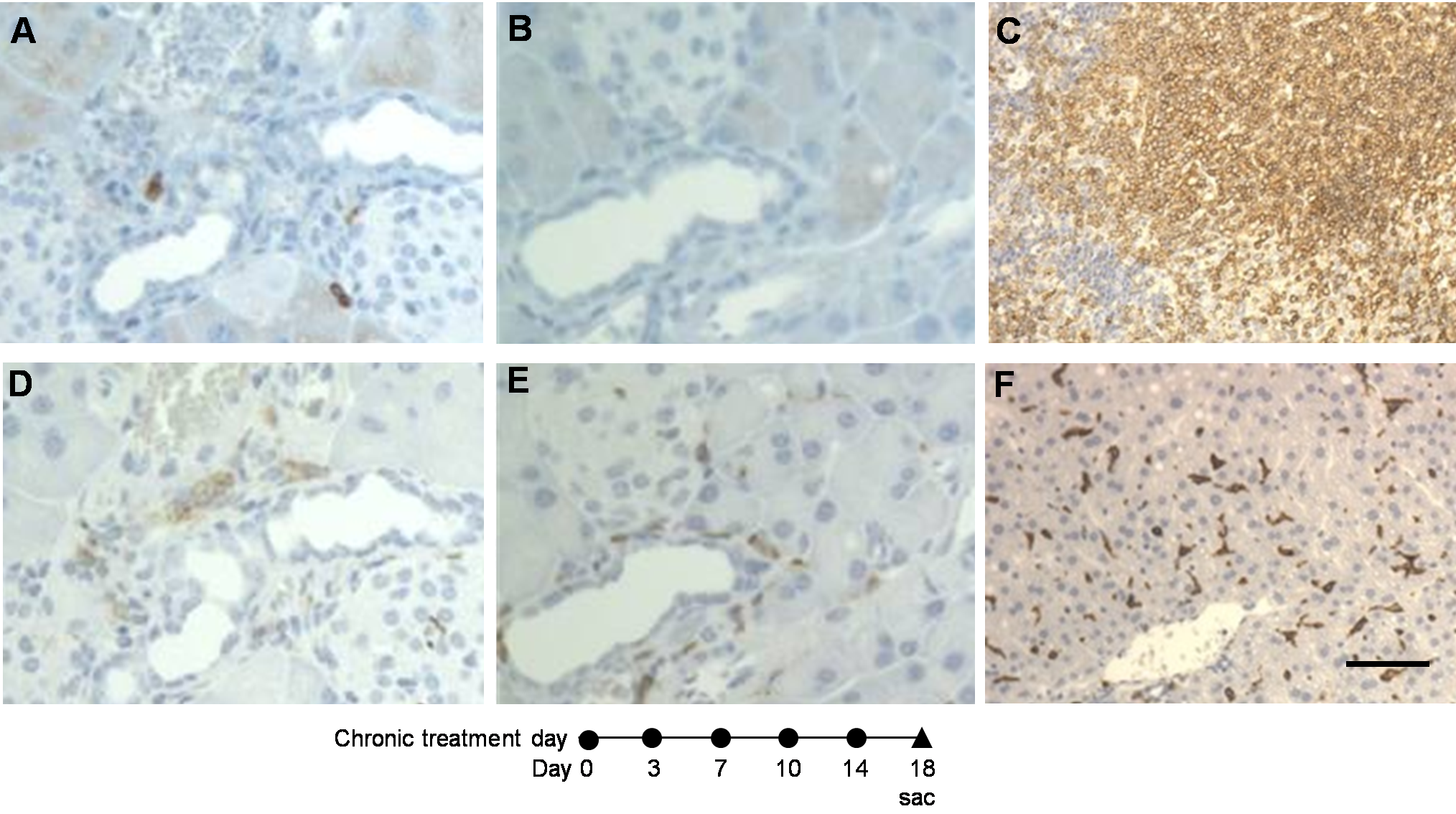

Supplement: Figure S3 — TWEAK treatment does not induce generalized pancreatic inflammation. Serial sections of pancreas from normal adult mice treated twice weekly with control (A, D) or TWEAK (B, E) immunostained for CD3 (A, B) and F4/80 (D, E) on day 18 after treatment. Positive control staining in spleen for CD3 staining (C) and in liver for F4/80 staining (F). Scale bar = 50 μm. (TIFF) [file pone.0072132.s003.tif]

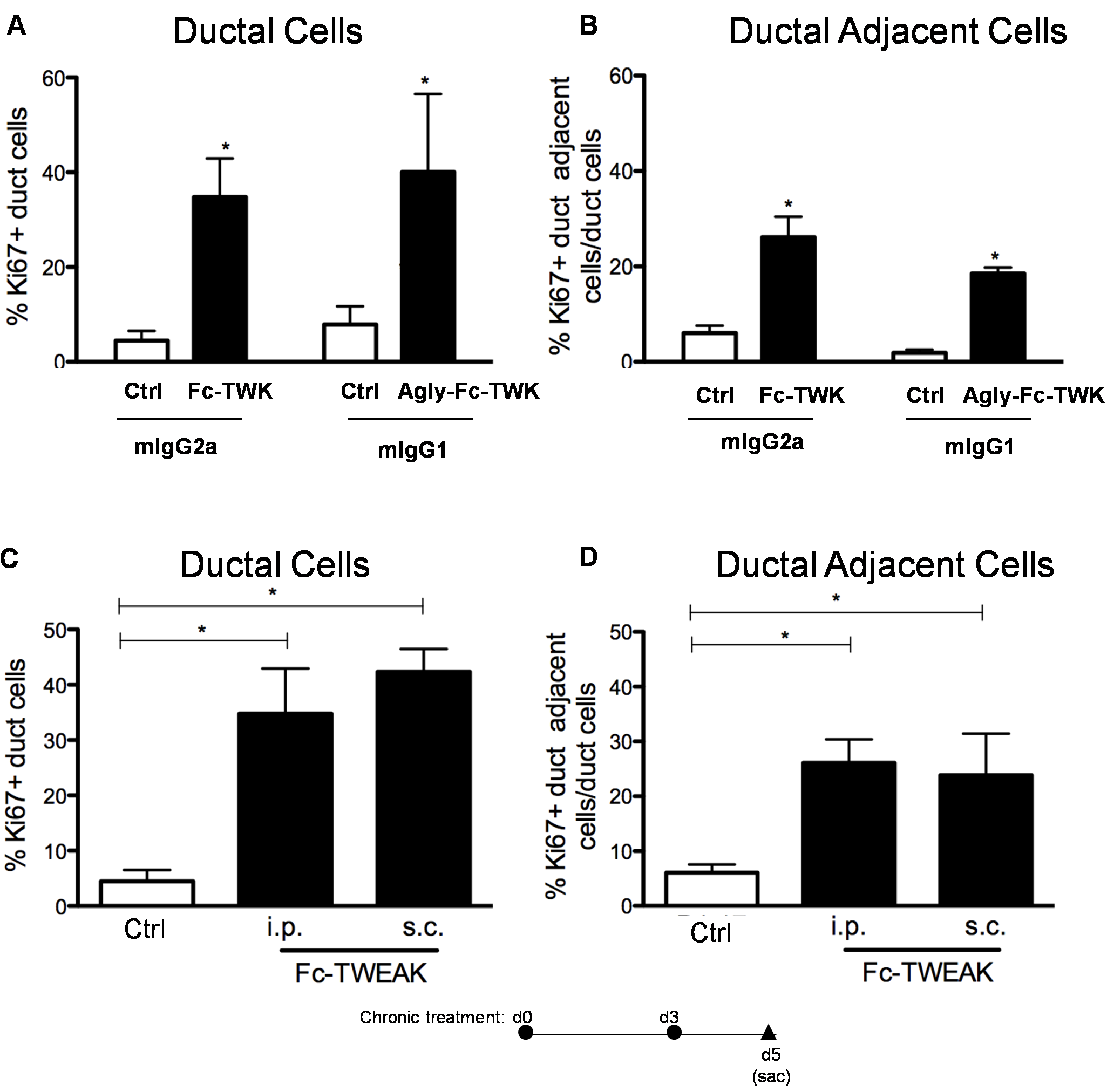

Supplement: Figure S4 — TWEAK-induced duct cell proliferation does not involve Fc-effector function nor is caused by peritonitis. (A–B) Quantification of % Ki-67+ duct (A) and duct adjacent cells (B) per total duct cells in pancreas from normal adult mice at day 5 after Ctrl mIgG2a (P1.17, control for Fc-TWEAK), Fc-TWEAK, Ctrl mIgG1 (1E6, (control for Agly-Fc-TWEAK) or Agly-Fc-TWEAK twice weekly. Controls (white bars); TWEAK treated (black bars). (C–D) Quantification of the % Ki-67+ duct (C) and duct adjacent cells (D) per total duct cells in pancreas at day 5 after Crtl (control Ig P1.17) or Fc-TWEAK subcutaneously (s.c.) or intraperitoneally (i.p.). Controls (white bars); TWEAK treated (black bars). Data are shown as mean±SEM (n = 4); * P<0.05 for TWEAK treatment vs control. (TIFF) [file pone.0072132.s004.tif]

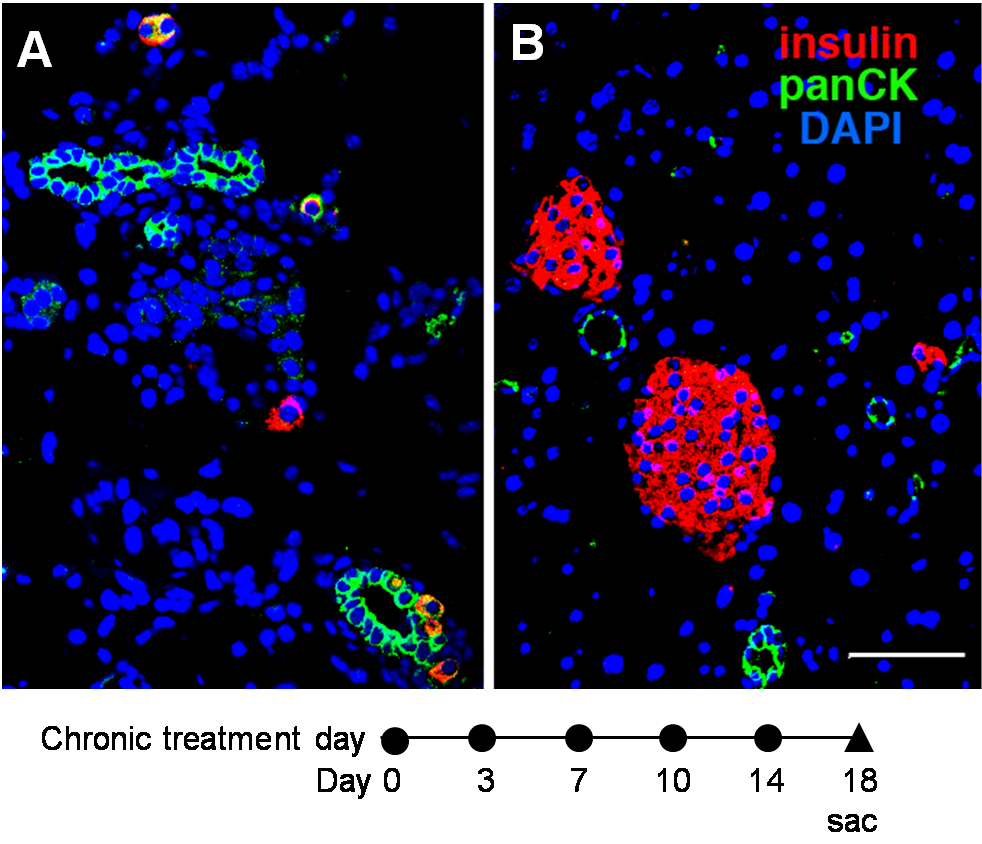

Supplement: Figure S5 — Co-expression of insulin and pan-cytokeratin in some cells of the focal ductal structures after chronic TWEAK treatment. Immunofluorescent staining of pancreas at day 18 after chronic TWEAK treatment (A) shows some insulin positive cells (red) also express panCK (green) (coexpression- yellow/orange) in the complex ductal structures but the Ig controls (B) do not have these regions and have no co-expression of the insulin and panCK. Magnification bar = 50 µm. (TIFF) [file pone.0072132.s005.tif]

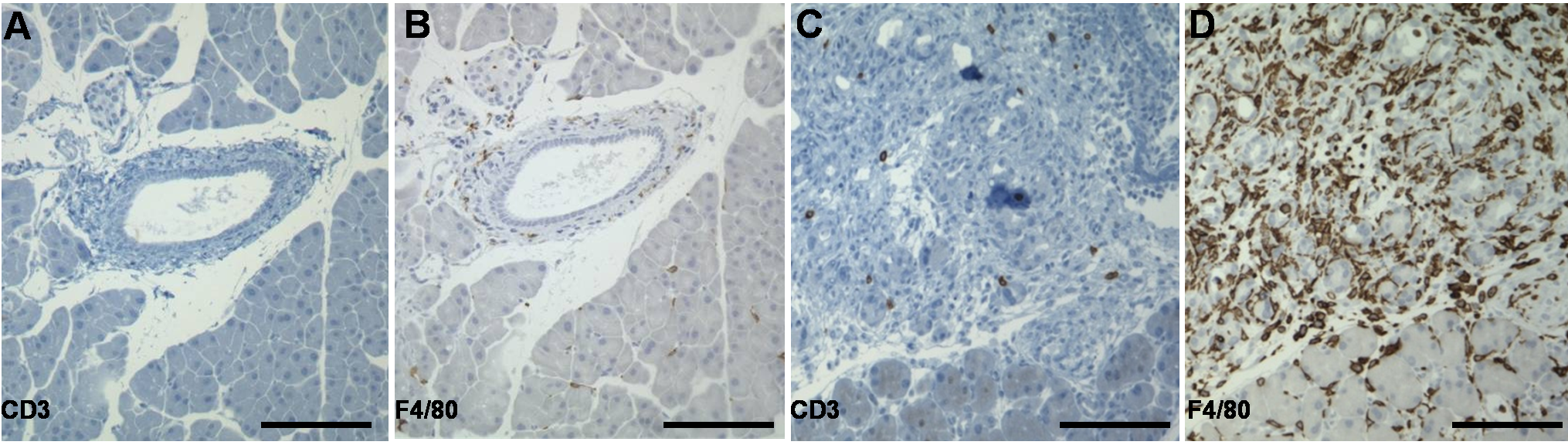

Supplement: Figure S6 — Inflammatory infiltrates were recruited to the regenerating foci after Px. Serial sections from sham-operated (A, B) and Px at 4 days post surgery (C, D) were immunostained for CD3 (A, C) and F4/80 (B, D). Scale bar = 200 μm. (TIFF) [file pone.0072132.s006.tif]
